# Dual graph convolutional neural network for predicting chemical networks

**DOI:** 10.1186/s12859-020-3378-0

**Published:** 2020-04-23

**Authors:** Shonosuke Harada, Hirotaka Akita, Masashi Tsubaki, Yukino Baba, Ichigaku Takigawa, Yoshihiro Yamanishi, Hisashi Kashima

**Affiliations:** 1 Kyoto University, Kyoto, 6068501 Japan; 2 Preferred Networks, Tokyo, 1000004 Japan; 3 National Institute of Advanced Industrial Science and Technology, Tokyo, 1350064 Japan; 4 Tsukuba University, Ibaraki, 3058577 Japan; 5 Hokkaido University, Hokkaido, 0600808 Japan; 6 Kyushu Institute of Technology, Fukuoka, 8208502 Japan; 7 Riken AIP, Tokyo, 1030027 Japan

**Keywords:** Chemical network prediction, Graph convolutional neural network, Graph of graphs

## Abstract

**Background:**

Predicting of chemical compounds is one of the fundamental tasks in bioinformatics and chemoinformatics, because it contributes to various applications in metabolic engineering and drug discovery. The recent rapid growth of the amount of available data has enabled applications of computational approaches such as statistical modeling and machine learning method. Both a set of chemical interactions and chemical compound structures are represented as graphs, and various graph-based approaches including graph convolutional neural networks have been successfully applied to chemical network prediction. However, there was no efficient method that can consider the two different types of graphs in an end-to-end manner.

**Results:**

We give a new formulation of the chemical network prediction problem as a link prediction problem in a graph of graphs (GoG) which can represent the hierarchical structure consisting of compound graphs and an inter-compound graph. We propose a new graph convolutional neural network architecture called dual graph convolutional network that learns compound representations from both the compound graphs and the inter-compound network in an end-to-end manner.

**Conclusions:**

Experiments using four chemical networks with different sparsity levels and degree distributions shows that our dual graph convolution approach achieves high prediction performance in relatively dense networks, while the performance becomes inferior on extremely-sparse networks.

## Background

Predicting chemical networks, consisting of a set of interactions among chemical compounds, is one of the fundamental tasks in bioinformatics and chemoinformatics, as well as predicting chemical properties of each compound. Large-scale analysis of chemical networks is useful for metabolic engineering [1–5] and various applications in drug discovery [6–12]. The rapid growth of the amount of available data including chemical structures and networks has enabled applications of data-driven approaches such as statistical modeling and machine learning methods [13]. Chemical compounds and chemical networks are often modeled as graphs which are general and powerful data representations of complex real-world phenomena. In a molecular compound graph, the nodes correspond to atoms and the edges correspond to chemical bonds among them. A chemical network is also described as a graph over compounds, where the nodes correspond to compounds and the edges correspond to chemical interactions between them.

Molecular fingerprinting [14] is a widely used way for molecular graph representation, where each compound is represented as a fixed-dimensional feature vector. Each element of a molecular fingerprint corresponds to a substructure (e.g., benzene ring) and a chemical property (e.g., aromatic). Examples include PubChem fingerprint [15], Extended-connectivity fingerprint [16], E-State fingerprint [17], and MACCS fingerprint [18]. They have been used for predicting various chemical properties, but the performance depends heavily on the choice of fingerprints. Statistical machine learning methods such as kernel methods have also been successfully applied to chemical property prediction [19–21]. In addition, statistical machine learning methods have been applied to predicting chemical networks such as metabolic reactions [22–27], drug-drug interactions [28–31] and beneficial drug combinations [32, 33] by taking a pair of compounds as an input to a classifier.

Most of the above mentioned studies are based on off-the-shelf feature representation of chemical compounds such as the molecular finger printings and tailored similarity functions such as kernel functions. More recently, driven by the significant advances of deep neural networks, researchers are moving to automatic extraction of flexible and expressive compound features from data, which succeed in improving the predictive performance [34]. Typical studies consider chemical property prediction formulated as classification or regression problems based on representation learning of compounds such as graph convolutional neural networks [35–39]. Some studies predict chemical networks by taking compound pairs as inputs [40]. Although not necessarily being specific to chemical network prediction, representation learning from chemical networks is mainly based on network embedding methods [41–43].

**Fig. 1:**
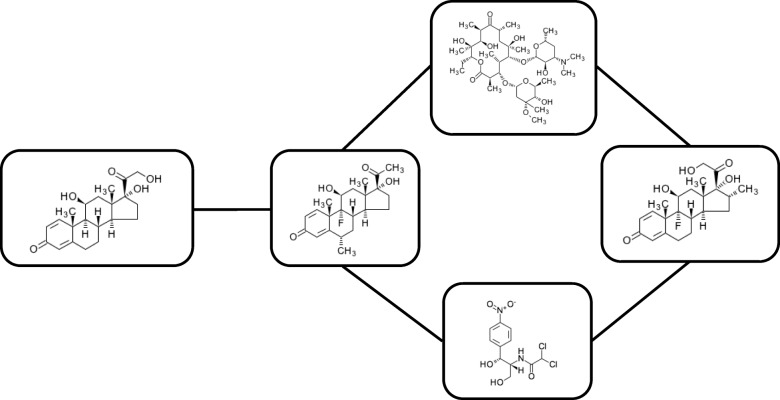
Chemical network. A chemical network is represented as a graph of graphs consisting of an external graph and a set of internal graphs. Each node of the external graph corresponds to a chemical compound, and each compound has its own internal graph structure representing chemical bonds among its atoms

Most of the previously mentioned studies represent both chemical compounds and their interaction networks as graph structured data. Despite the wide ranging and rapidly spreading applications of deep learning in the chemical domain, chemical compound graphs and their interaction networks have been studied rather independently. Such different-level structures in a chemical network are unified as a hierarchically-structured graph, namely, a graph of graphs (GoG) (Fig. 1). This hierarchically structured graph has two types of graph structures: the internal graph structure inside a single compound and the external graph structure among a set of compounds.

In this paper we develop an effective modeling method for the GoG which has a more general and complex graph structure than a single graph, and to consider the link prediction task on a GoG. We extends the existing graph convolutional neural network to GoGs by introducing a new architecture called *dual graph convolutional neural network*, which allows us to (i) seamlessly handle both internal and external graph structures in an end-to-end manner using backpropagation [44] and (ii) efficiently learn low-dimensional representations of the GoG nodes. We conduct experiments of the link prediction task using four chemical network datasets, that are, drug-drug interaction network, drug indication network, drug function network, and metabolic reaction network. They have different levels of sparsity and different tail weights of degree distributions, and we use them for evaluating applicability of the proposed approach.

## Method

We formulate the chemical network prediction problem as a link prediction problem in a graph of graphs (GoG). Our solution which we call dual graph convolution is an extension of the graph convolutional neural networks that enables us end-to-end modeling of chemical networks using two kinds of graph convolution layers: internal graph convolution layers and external graph convolution layers.

### Problem formulation

Throughout the paper, we denote vectors by bold lowercase letters (e.g., $\mathbf {v} \in \mathbb {R}^{d}$), matrices by bold uppercase letters (e.g., $\mathbf {M} \in \mathbb {R}^{m \times n}$), and scalars and discrete symbols (such as graphs and nodes) by non-bold letters (e.g., $\mathcal G$ and *n*).

A GoG is a hierarchically structured graph $\mathcal G = (\mathcal V, \mathcal A)$, where $\mathcal V$ is the set of nodes, $\mathcal A$ is the adjacency list. Each node in the GoG is also a graph, which we denote by $G = (V, A) \in \mathcal V$, where *V* is the set of nodes, and *A* is the adjacency list. We refer to $\mathcal G$ as an *external graph* and *G* as an *internal graph*. Generally, a GoG can have more than two levels. In this paper, we only consider two levels for simplicity, and refer to them by internal graph and external graph; however, our fundamental idea itself is easily generalized to GoGs with more levels. A chemical network is represented as a GoG $\mathcal G$, whose nodes $\mathcal V$ are the set of compounds, and whose edges referred to by its adjacency list $\mathcal A$ are the set of binary relations (e.g., interact or not) among the compounds. For each compound $G = (V, A) \in \mathcal V$, *V* is the set of the atoms included in the compound, and *A* indicates the set of chemical bonds among the atoms.

Given a GoG, our goal is to obtain a feature representation of each internal graph $G \in \mathcal V$ and to predict the probability of the existence of a (hidden) link between arbitrary two internal graphs $G_{i}, G_{j} \in \mathcal V$.

### Proposed method: dual graph convolutional neural network

**Fig. 2:**
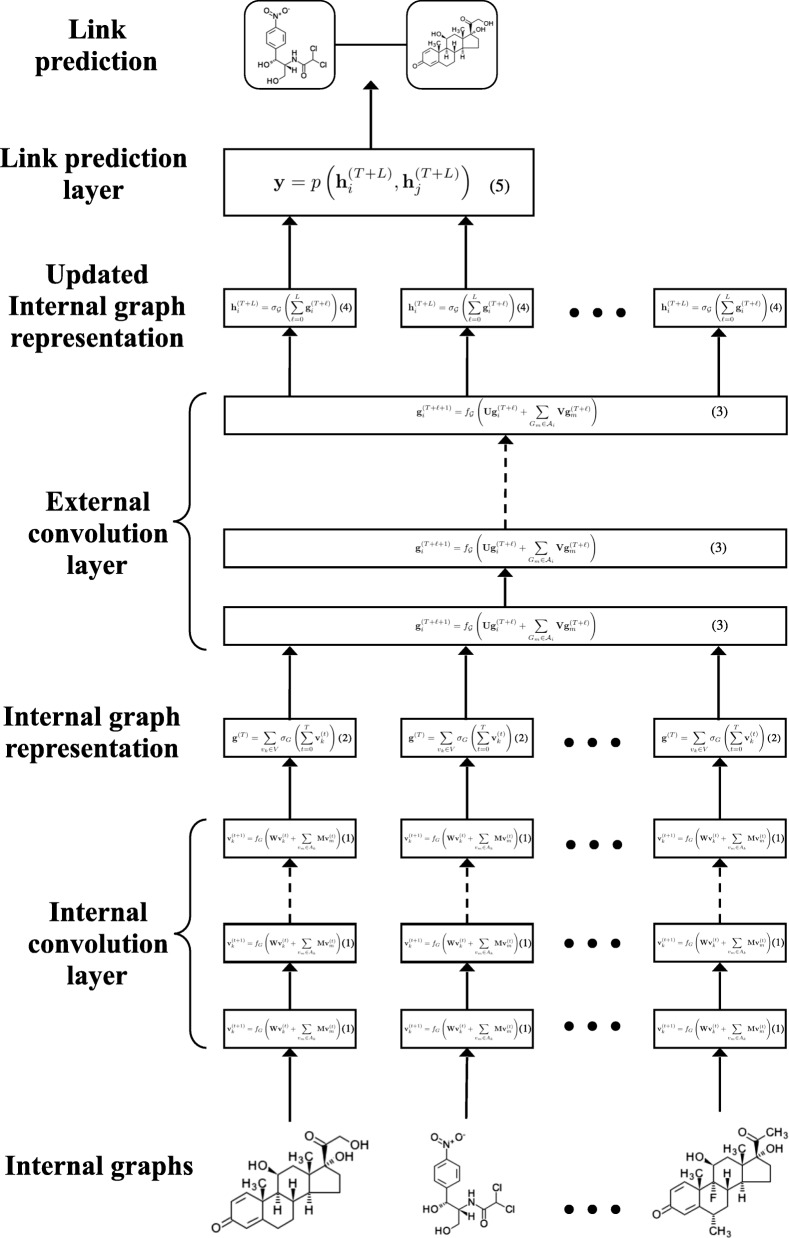
Dual graph convolutional neural network. The internal convolution layer extracts features from the molecular compound graphs, which are followed by the external convolutions layer to incorporate structural information of the inter-compound network

We propose the *dual graph convolutional neural network* for a GoG that consists of three components (Fig. 2): the internal graph convolution layer (“Internal graph convolution” section), the external graph convolution layer (“External graph convolution” section), and the link prediction layer (“End-to-end training of the link prediction function”).

#### Internal graph convolution

The internal convolution layer takes a chemical compound represented as an internal graph *G*=(*V*,*A*) as its input, and outputs a fixed-dimensional vector representation for the compound. At the bottom of the internal convolution layer, the low-dimensional real-valued vector representation $\mathbf v_{k} \in \mathbb R^{d}$ for the *k*-th atom *v*_*k*_∈*V* is randomly initialized, where *d* is the dimension of the vector. Each **v**_*k*_ is initialized differently depending on the types of atoms (e.g., hydrogen or oxygen), and trained using back-propagation as well as the subsequent external convolution and link prediction layers in an end-to-end manner (“Drug indication network” section).

Given the initialized atom feature **v**_*k*_ for each atome *v*_*k*_, starting from $\mathbf v_{k}^{(0)} = \mathbf v_{k}$, we update ${\mathbf v}_{k}^{(t)}$ to ${\mathbf v}_{k}^{(t+1)}$ by the *internal convolution* operation:

1 $$\begin{array}{@{}rcl@{}}  \mathbf v_{k}^{(t+1)} = f_{G} \left(\mathbf W \mathbf v_{k}^{(t)} + \sum_{v_{m} \in A_{k}} \mathbf M \mathbf v_{m}^{(t)} \right), \end{array} $$

where *f*_*G*_ is the non-linear activation function such as ReLU. *A*_*k*_ is the list of the adjacency atoms of *v*_*k*_, and $\mathbf W \in \mathbb R^{d \times d}$ and $\mathbf M \in \mathbb R^{d \times d}$ are the weight matrices to be learned. As with the graph convolution of Duvenaud et al. [35], each atom gradually incorporate global information of the compound graph into its representation by iterating the internal convolution step using the representations of its adjacent atoms. We make *T* iterations to obtain ${\mathbf v}_{k}^{(1)}, {\mathbf v}_{k}^{(2)}, \dots, {\mathbf v}_{k}^{(T)}$.

Finally, summing all of the atom features over all of the internal convolution steps to obtain the compound representation as

2 $$\begin{array}{@{}rcl@{}}  \mathbf g^{(T)} = \sum_{v_{k} \in V} \sigma_{G} \left(\sum_{t=0}^{T} \mathbf v_{k}^{(t)}\right), \end{array} $$

where *σ*_*G*_ is a non-linear function such as the softmax function. We denote by $\mathbf g_{i}^{(T)}$ the representation of compound graph $G_{i} \in \mathcal V$, which will be the initial feature vector in the external graph convolution introduced in “External graph convolution” section.

We have freedom of choices for the nonlinear activation functions and parameter initialization. In the experiments, we use the ReLU function as activation function *f*_*G*_. We use different **W** and **M** for different degrees (|*A*_*k*_| and |*A*_*m*_|) and convolutional steps. We ignored the chemical bond types mainly for computational efficiency; the data size is increased by encoding the bond information as adjacency matrices. This is compensated to some extent by introducing the different parameter matrices for different node degrees by following Duvenaud et al. ([35]). The representation **v**_*k*_ of atom *k* is randomly initialized using a Gaussian distribution depending on the atom type, the valence, the number of hydrogen, the number of degrees, and the aromatic sign as with the neural finger print [35]. We use the softmax function as *σ*_*G*_ in Eq. (2).

#### External graph convolution

The set of representations for all the compound graphs $\{ \mathbf g_{i}^{(T)} \}_{G_{i} \in {\mathcal V}}$ are further updated with the *external convolution* to incorporate structural information of the external chemical network. Starting from *ℓ*=0, we make *L* updates using the external convolution operation given as

3 $$\begin{array}{@{}rcl@{}}  \mathbf g_{i}^{(T+\ell+1)} = f_{\mathcal G} \left(\mathbf U \mathbf g_{i}^{(T+\ell)} + \sum_{G_{m} \in \mathcal A_{i}} \mathbf V \mathbf g_{m}^{(T+\ell)} \right), \end{array} $$

where $f_{\mathcal G} $ is a non-linear activation function, $\mathcal A_{i}$ is the adjacency list of compound *G*_*i*_ in the external chemical network, and $\mathbf U \in \mathbb R^{d \times d}$ and $\mathbf V \in \mathbb R^{d \times d}$ are the weight matrices to be learned. We obtain the final chemical graph representation ${\mathbf h}_{i}^{(T+L)}$ considering all of the *L* external convolution steps as

4 $$\begin{array}{@{}rcl@{}}  \mathbf h_{i}^{(T+L)} = \sigma_{\mathcal G} \left(\sum_{\ell=0}^{L} \mathbf g_{i}^{(T+\ell)}\right), \end{array} $$

where $\sigma _{\mathcal G}$ is a non-linear activation function; we use the softmax function in our experiments. Note that dual convolution does not aim to obtain a single representation of the external chemical network, but to obtain the representation of each compound considering both the internal and external graph structures, which will be used in the following link prediction layer.

We use the softmax function as $f_{\mathcal G}$, and use different **U** and **V** for different convolutional steps. we do not distinguish different degrees because the interaction networks have much larger degrees than molecular graphs.

#### End-to-end training of the link prediction function

The link between two compounds *G*_*i*_ and *G*_*j*_ is predicted using their final representations $\mathbf h_{i}^{(T+L)}$ and $\mathbf h_{j}^{(T+L)}$. A multi-layer neural network *p* outputs a two-dimensional vector $\mathbf y \in \mathbb R^{2}$:

5 $$\begin{array}{@{}rcl@{}}  \mathbf y = p \left(\mathbf h_{i}^{(T+L)}, \mathbf h_{j}^{(T+L)} \right), \end{array} $$

and the softmax function gives the final link probability:

6 $$ p_{t} = \frac{\exp(y_{t})}{\sum_{k} \exp(y_{k})},  $$

where *t*∈{0,1} is the binary label (i.e., link or no-link).

We use the two-layer neural network as the link prediction network (5) whose input is given as

7 $$ \left(\mathbf h_{i}^{(T+L)}+ \mathbf h_{j}^{(T+L)} \right)\oplus\left(\mathbf h_{i}^{(T+L)} \odot \mathbf h_{j}^{(T+L)}\right),  $$

where ⊕ is the concatenation of two vectors and ⊙ is the Hadamard product (i.e., element-wise product). Note that the symmetry of *p* with respect to its two inputs is ensured because the above construction is symmetric with respect to $\mathbf h_{i}^{(T+L)}$ and $\mathbf h_{j}^{(T+L)}$. We use ReLU for all of the non-linear activation functions.

Given a set of all compound graphs and some observed links among them as the training dataset, we minimize the cross-entropy loss function:

8 $$ \mathcal L(\Theta) = -\sum_{i=1}^{N} \log p_{t_{i}}  $$

with respect to the model parameters *Θ* including the set of all weight matrices in the dual graph convolutional network and the atom features (that are initialized randomly). *N* is the total number of internal graph pairs in the training dataset, and *t*_*i*_ is the *i*-th label (link or no-link).

## Result

We evaluate the proposed dual graph convolution that combines the structural information of both internal and external graphs in a GoG. We compare the link prediction accuracy of the proposed method and several baselines using four chemical networks. The experimental results show the proposed method works well for moderately dense chemical networks with heavy-tailed degree distributions. In an extremely sparse and light-tailed network, inter-compound links are almost useless, and the domain specific features (i.e., Morgan indices) perform the best. The internal convolution also suffers from the lack of inter-compound links used as the training data.

### Datasets

**Fig. 3:**
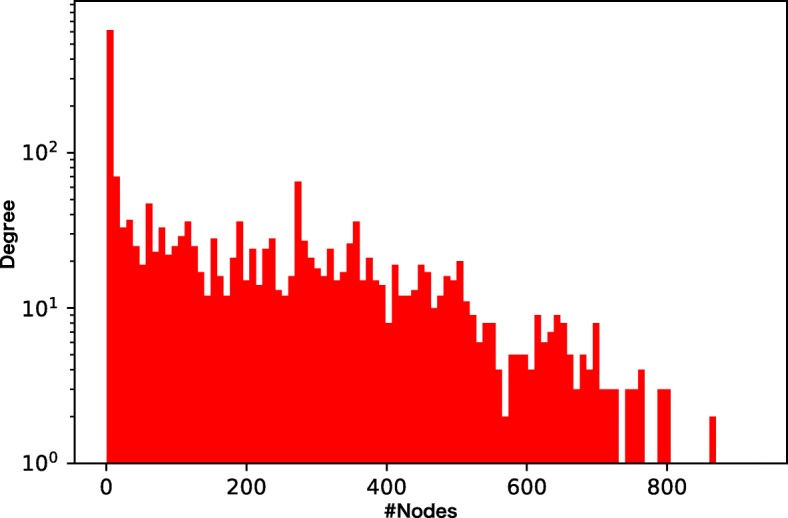
Node degree distribution of the drug-drug interaction network. The network is dense and very heavy-tailed

**Fig. 4:**
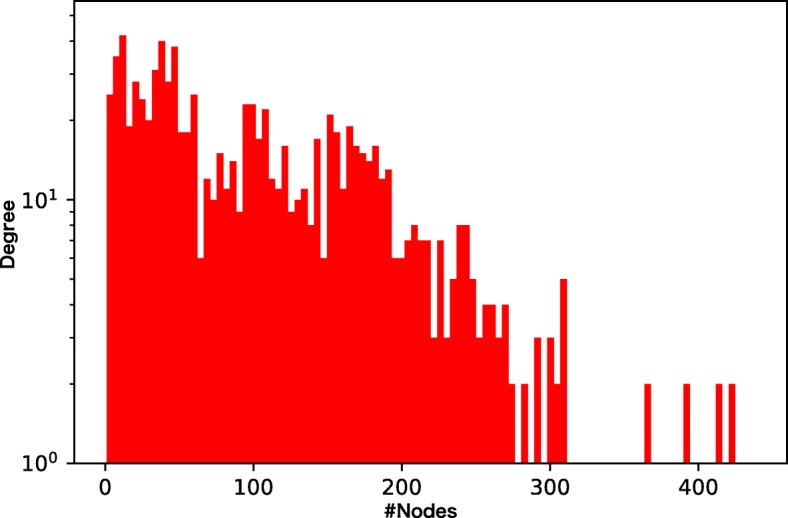
Node degree distribution of the drug indication network. The network is dense and heavy-tailed

**Fig. 5:**
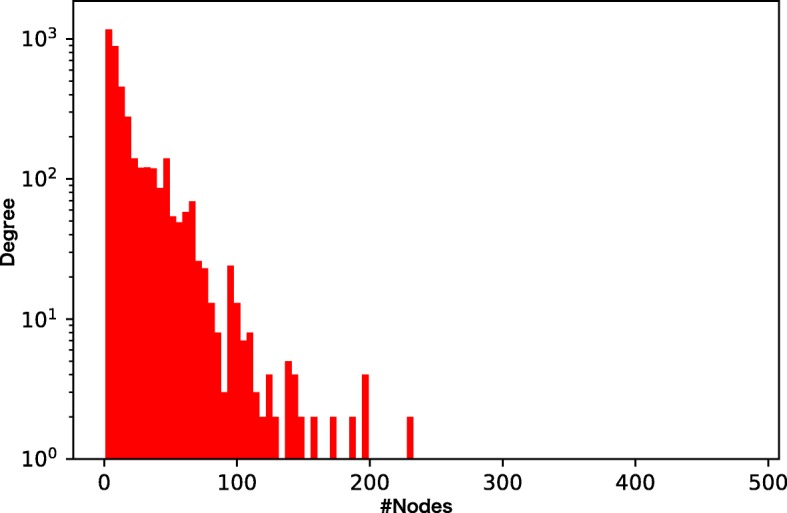
Node degree distribution of the drug function network. The network is sparse and light-tailed

**Fig. 6:**
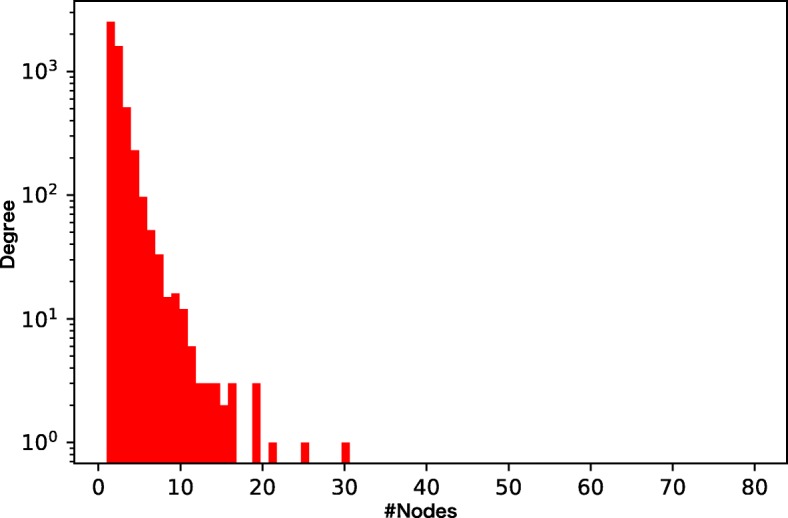
Node degree distribution of the metabolic reaction network. The network is extremely sparse and light-tailed

We prepare four different chemical GoGs with different levels of sparsity and different weights of the tails of the degree distributions (Figs. 3, 4, 5, and 6). Among the four chemical networks we describe below, the first two have heavy-tailed degree distributions, while the others have relatively light-tailed. One of our main interests is to obtain insights about the conditions of chemical networks in which our proposed neural network architecture is effective.

#### Drug–drug interaction network

The first dataset is a drug–drug interaction network that is a network of drug compounds where two compounds are connected with a link if they are known to interact, interfere, or cause adverse reactions when taken together.

We used 1,993 approved drugs that have at most 64 atoms in DrugBank database (blackhttps:// www.drugbank.ca/releases/latest), version 5.0.9 (as of October 2, 2017) [45]). Out of all possible $\binom {1993}{2}=1,985,028$ compound pairs, 186,555 have edges; the link density is 0.0940 which means it is a relatively dense network.

We have only positive links in this dataset; this situation is sometimes dealt with positive-and-unlabeled learning [46]; however, we just regard sampled no-links as the negative links for simplicity [47]. We randomly choose *n* positive links and *n* no-links (i.e., negative links) as the training dataset. We vary *n* from 1k to 10k to investigate the importance of incorporating the information of the external graph by the external convolution. As the test dataset, we randomly extract positive and negative links from the same data distribution as the original network to preserve the data imbalance, which results in 9,398 positive links and 90,601 negative links.

#### Drug indication network

The drug indication dataset is a network of drug compounds where two compounds are linked if they have similar indications. Our dataset is extracted from SIDER2, version 4.1 (as of October 21, 2015, http://git.dhimmel.com/SIDER2/), which includes 938 drugs that have fewer than 64 atoms. Out of all possible $\binom {938}{2}=439{,}453$ compound pairs, we define 48,679 positive links whose indication values are positive. As well as the drug-drug interaction network, we sample no-links as the negative links. We use 2,215 positive links and 17,785 negative links as the test set.

#### Drug–function network

The Drug function network dataset is a network of drug compounds where two compounds are linked if they share a same target protein. From the original dataset [48] which uses the DrugBank database, version 2.5 (as of January 29, 2009), we used 3,918 compounds that have fewer than 64 atoms. Out of all possible $\binom {3918}{2}=7,673,403$ compound pairs, 35,562 have edges; the link density is 0.0046 which means it is a sparse network.

As well as the drug-drug interaction dataset, this network also has only positive links; therefore, we sample no-links as the negative links. We have 1,390 positive links and 298,609 negative links in the test set.

#### Metabolite reaction network

The last dataset is the metabolic reaction network dataset that is a network of metabolite compounds where two compounds are linked if they are the substrate-product pair in an enzymatic reaction on metabolic pathways [26]. Enzymatic reactions and the associated chemical compounds were obtained from the KEGG LIGAND database, Release 62.0 [49]. In this study we collected 5,920 compounds that have fewer than 64 atoms. Out of all possible $\binom {5920}{2}=17,520,240$ compound pairs, only 5,041 have edges; the link density is 0.0003 which means it is an extremely sparse network. These edges are regarded as positive links, and the other compound-compound pairs are regarded as negative links.

Different from the other datasets, this network has both 5,041 positive links and 220,096 negative links; the test set consists of 223 positive links 9,777 negative links.

### Specific implementation of the proposed model

We implement the proposed dual graph convolutional network using Chainer [50] and use ADAM [51] as the optimizer. The learning rate is set to 0.001. We use held-out development datasets to choose *d*, the number of dimension of the internal graph representations, from {32,62,128}, and the numbers of convolution steps *T* and *L* from {1,3,5}. Similarly, the batch size is selected from {64,128,256}. Generally, especially in dense external networks, the number of external convolution seems more important than that of the internal convolution. We also set the dropout rate 0.2 in Eq. (1). The sizes of the two layers in the link prediction function are set to 128 and 64, respectively.

### Baseline methods

We compare the dual graph convolutional network with several baselines, namely, (i) a model using only internal graph convolution, (ii) models based only on external graph structures, (iii) a model based on hashed Morgan fingerprints instead of the internal graph convolution, and (iv) several similarity indices for link prediction.

#### Internal graph convolution

Internal graph convolution obtains 64-dimensional representations of molecular graphs. We do not use the inter-compound network, and we create a feature vector for each molecule by the internal convolution and directly use it as an input to the link prediction network. We use the same convolution formula as that by Duvenaud et al. [35].

#### External graph embedding

External graph embedding is a standard approach to link prediction using only the inter-compound network (i.e., the external graph). We test DeepWalk [41] that is one of the well-known embedding methods, and also test the general relational embedding model proposed by Yan et al. [52] where the latent representation for each molecule is initialized to a 64-dimensional random vector. The link prediction network (5) is applied to a pair of molecules.

#### Hashed morgan fingerprints

We use the hashed Morgan fingerprints, which is well-known off-the-shelf chemical features based on chemical substructures. We use 2048-dimensional Morgan fingerprints as a feature vector of a molecule. The link prediction network (5) is applied to a pair of molecules.

#### Similarity indices

A similarity index gives the similarity of arbitrary two nodes in a graph. Typical similarity indices include common neighbors index (CN), Jaccard’s coefficient index (Jaccard), and the Katz index (Katz). Table 1 summarizes their definitions. Despite their simplicity, they are quite powerful for biological network prediction [53]. Links are predicted in descending order of their similarity scores.

**Table 1:** Definitions of several similarity indices between two nodes (*G*_*i*_,*G*_*j*_) in a GoG $\mathcal {G}$

Similarity index	Definition
Common neighbours	$ \mid \mathcal {N}(G_{i})\cap \mathcal {N}(G_{j})\mid $
Jaccard’s coefficient	$\frac {\mid \mathcal {N}(G_{i})\cap \mathcal {N}(G_{j})\mid } {\mid \mathcal {N}(G_{i})\cup \mathcal {N}(G_{j})\mid } $
Katz	$(I-\beta \boldsymbol {\mathcal {A}})^{-1}-I$

$\mathcal {N}(G)$ indicates the neighbor set of node *G*. *I* is the identity matrix and ${\mathcal {A}}$ is the adjacency matrix of $\mathcal {G}$. *β* is the constant parameter that controls path weights depending on their lengths, and we set *β*=0.001

### Results

All the datasets we use have imbalance nature in terms of the number of positive and negative links; therefore we measure the predictive performance of each method using (i) ROC-AUC which is not affected by the label imbalance and (ii) PR-AUC which can suitably evaluate the performance on imbalanced datasets.

**Fig. 7:**
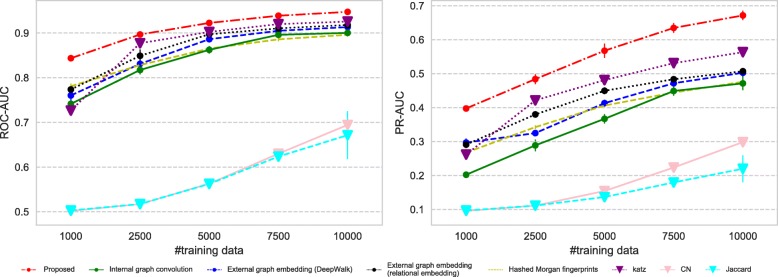
Prediction performance for the drug-drug interaction network. The performance is given in both ROC-AUC (left) and PR-AUC (right). The proposed dual graph convolution method performs well for this dense network with the very heavy-tailed degree distribution

**Fig. 8:**
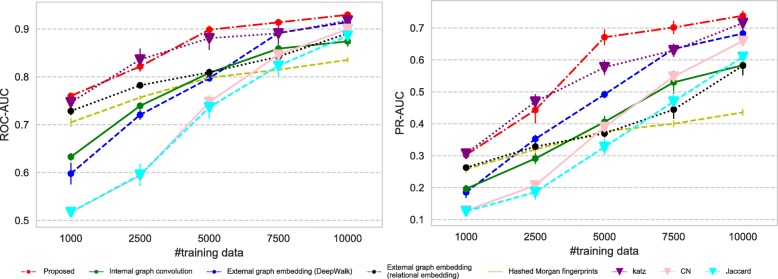
Prediction performance for the drug indication network. The performance is given in both ROC-AUC (left) and PR-AUC (right). The proposed dual graph convolution method performs well for this dense network with the heavy-tailed degree distribution

**Fig. 9:**
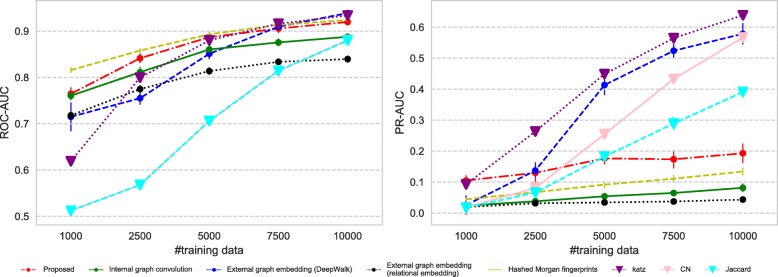
Prediction performance for the drug function network. The performance is given in both ROC-AUC (left) and PR-AUC (right). The advantage of the proposed method is limited for this sparse network with the light-tailed degree distribution

**Fig. 10:**
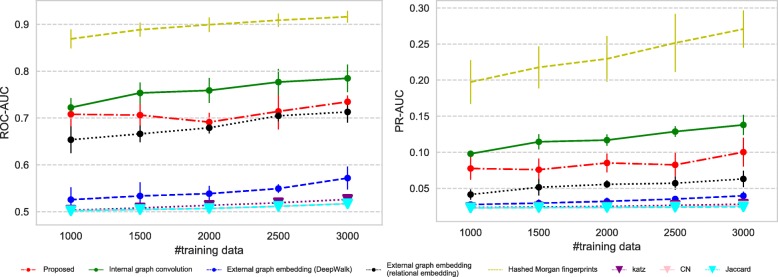
Prediction performance for the metabolic reaction network. The performance is given in both ROC-AUC (left) and PR-AUC (right). The proposed method shows the limited performance for this extremely sparse network with the light-tailed degree distribution. Inter-compound links are almost useless as features, and therefore the domain specific features (i.e., Morgan indices) perform the best. The internal convolution also suffers from the lack of the links as the training data

Figures 7, 8, 9, and 10 show the comparison of the proposed method and the four baselines in terms of ROC-AUC and PR-AUC with different training set sizes. In Fig. 7 and Fig. 8, the dual graph convolution network achieves consistently better ROC-AUC and PR-AUC scores over the baselines in the drug-drug interaction network and the drug indication network. This is probably due to the high density and the heavy-tailed degree distribution of its external graph (i.e., inter-compound graph). In such networks, the external links are likely to efficiently connect many nodes with short paths, and therefore, the dual convolution successfully extracts structural features in the external graph.

Figure 9 shows the result for the drug function network. The advantage of the dual convolution is rather limited in the relatively sparse light-tailed network, because the efficiency of external node connections is lower than the previous networks. Interestingly the performance of DeepWalk and the similarity indices, especially, the Katz index, improves as the size of the training set increases; this implies that DeepWalk and the Katz index successfully extracts structural features from longer paths. Given that DeepWalk the similarity indices do not consider the internal graph structure at all, information of the inter-compound network seems more crucial than the compound graphs in the drug-function network.

In contrast to the other networks, the metabolite network is an extremely sparse that has very few inter-compound links and a very light-tailed degree distribution. The inter-compound links are almost useless in this network, and therefore the relational embedding method, DeepWalk and the similarity indices that solely depend on inter-compound links perform poorly (Fig. 10). Especially, the performance of DeepWalk and the Katz index significantly degrades in terms of both ROC-AUC and PR-AUC, because both are based on paths on a graph, and they cannot “walk" over the inter-compound links in such a sparse network. Similarly, the proposed method cannot even benefit from the external convolution, and it suffers from the sparsity of the network. The lack of the external links as the training dataset is also a severe limitation for extracting features from the internal graphs. In such a sparse data domain, traditional off-the-shelf features such as Morgan indices are still reliable choices.

In summary, our experimental results suggest that the dual convolution architecture is effective for relatively dense networks, especially when both the internal and external structures must be considered in an integrated manner. Among the networks, the links of the drug-drug interaction network represent direct chemical interactions between two compounds. In such networks, non-trivial combination of different chemical substructures of both ends of a link contributes to the interaction.

## Discussion

We discuss the computational efficiency and extendability of the proposed model.

We compare the complexity and scalability of our dual convolution model and the existing graph neural network using only the internal graph convolution. Table 2 shows the comparison of complexity in terms of time and space required for one update of mini-batch backpropagation training. In terms of time complexity, while the internal graph convolution requires the linear complexity with respect to the number of nonzero elements |**A**| in the adjacency matrix of internal graph, our method suffers from the (linear) complexity depending on the numbers of nodes involved in the external graph convolution and the complexity of internal graph convolutions. In terms of space complexity, in addition to storing the external embeddings, we need to store the internal graph embeddings which are associated with each external node. Taking the overlapping of different nodes into account, both complexity can be less than the worst case *BD*^*L*^. However, this still leads to computational problems in terms of both time and space complexity. This is a limitation of the proposed method especially when we consider deeper convolutional network architectures, which is an important problem to be addressed in future work.

**Table 2:** Comparison of the time and space complexity of backpropagation training of the standard graph convolutional network (Internal convolution) and the proposed model (Dual convolution)

Method	Time complexity	Space complexity
Internal convolution [35]	*BT*|**A**|*d*+*BTN*_in_*d*^2^	*BTN*_in_*d*+*Td*^2^
Dual convolution (proposed)	$BD_{\text {ex}}^{L} d^{2}+D_{\text {ex}}^{L}(BT |\mathbf {A}| d+BTN_{\text {in}}d^{2})$	$BD_{\text {ex}}^{L}d+Ld^{2} + D_{\text {ex}}^{L}BTN_{\text {in}}d+Td^{2}$

We denote the dimension of each layer by *d*, which is fixed for all layers for simplicity. *N*_in_ are the average number of the nodes in the internal graphs, respectively. *B* is the batch size. *D*_ex_ is the average degree of an external graph. **A** is the adjacency matrix of *G*. |**A**| is the number of nonzero elements in **A**. We omit the complexity related to the multi-layer neural network and the memory of storing the graph for simplicity because the multi-layer neural network is common to the two models and storing the graph is basically not the crucial issue

We finally discuss the extendability of the proposed dual graph convolution model. What we proposed in this paper is a general graph neural network architecture for GoGs, and our proposed dual graph convolution is based on one of the simplest convolution operators [35]. Recent advances in graph neural networks have introduced various effective techniques such as graph attention [54], message passing [38], and neighbor sampling [55]. Most of these new techniques are independent of our proposed architecture and can be integrated into our architecture.

In this paper, we focused only on the link prediction problem on an inter-compound network, and we particularly designed the output layer for the specific problem. However, other tasks such as compound classification or clustering can also be addressed by replacing the final layer specialized for each specific task, which will be an interesting future work.

## Conclusion

We proposed a new formulation of the chemical network prediction problem as a link prediction problem in a GoG which can represent the hierarchical structure consisting of compound graphs and an inter-compound graph. We proposed a new graph convolutional neural network architecture called dual graph convolutional network that learns compound representations from both the compound graphs and the inter-compound network in an end-to-end manner. We demonstrated the effectiveness of the proposed method for predicting interactions among molecules by using four chemical GoGs. Our dual convolution approach achieved high prediction performance even though the features were lower-dimensional compared to the off-the-shelf features in relatively dense networks, while the performance becomes inferior on extremely-sparse external networks because of the difficulty of exploiting the information about the external networks.

## Data Availability

The datasets during the current study are available publicly and the source reference are given in main manuscript. The datasets during the current study are available from the corresponding author on reasonable request.

## Abbreviations

CN: Common neighbors index
GoG: Graph of graphs
Jaccard: Jaccard’s index
Katz: Katz index
